# MicroRNA-186-5p serves as a diagnostic biomarker in atherosclerosis and regulates vascular smooth muscle cell proliferation and migration

**DOI:** 10.1186/s11658-020-00220-1

**Published:** 2020-04-21

**Authors:** Bin Sun, Qingtao Cao, Meng Meng, Xiaolong Wang

**Affiliations:** 1 Department of Emergency Medicine, Yidu Central Hospital of Weifang, Weifang, 262500 Shandong China; 2 Department of Cardiovascular Medicine, Yidu Central Hospital of Weifang, Weifang, 262500 Shandong China; 3 Department of Emergency Cardiovascular Medicine, Weifang People’s Hospital, No. 151 Guangwen Street, Weifang, 261000 Shandong China

**Keywords:** MiR-186-5p, Atherosclerosis, Vascular smooth muscle cell, Proliferation, Migration

## Abstract

**Objective:**

MicroRNA dysregulation occurs in many human diseases, including atherosclerosis. Here, we examined the serum expression and clinical significance of miR-186-5p in patients with atherosclerosis, and explored its influence on vascular smooth muscle cell (VSMC) proliferation and migration.

**Methods:**

Blood samples were collected from 104 patients with asymptomatic atherosclerosis and 80 healthy controls. Quantitative real-time PCR was applied to measure the miR-186-5p level. An ROC curve was established to assess the discriminatory ability of the serum miR-186-5p level for identifying atherosclerosis from controls. CCK-8 and Transwell assays were used to evaluate the impact of miR-186-5p on cell behaviors.

**Results:**

Serum expression of miR-186-5p was significantly higher in atherosclerosis patients than in the control group. The serum miR-186-5p level showed a positive correlation with CIMT and could be used to distinguish atherosclerosis patients from healthy controls, with an area under the curve (AUC) score of 0.891. In VSMCs, overexpression of miR-186-5p significantly promoted cell proliferation and migration, while the opposite results were observed when miR-186-5p was downregulated.

**Conclusion:**

Overexpression of miR-186-5p has a certain diagnostic significance for atherosclerosis. Upregulation of miR-186-5p stimulates VSMC proliferation and migration. Therefore, it is a possible target for atherosclerosis interventions.

## Introduction

Atherosclerosis, a leading cause of cardiovascular diseases, is characterized by local thickening of the artery wall and plaque formation [1]. Although considerable progress has been made in its treatment, the risk of co-morbidities of atherosclerosis remains high. Atherosclerosis is a chronic and complex process involving various cellular and molecular changes [2].

Abnormal vascular smooth muscle cell (VSMC) behavior has been suggested to participate in its progression as VSMC proliferation and apoptosis are associated with the formation and vulnerability of atherosclerotic plaques [3, 4]. It is important to further explore the underlying molecular mechanisms involved in those pathophysiological process.

MicroRNAs (miRNAs) affect various biological processes by regulating target mRNA expression [5]. MiRNA dysregulation occurs in multiple human diseases, including atherosclerosis [6, 7], and the clinical values of these aberrantly expressed miRNAs are leveraged in the diagnosis and prognosis of human diseases [7, 8].

MiR-29b overexpression is detected in the serum of atherosclerosis patients, and its expression level is positively associated with carotid intima-media thickness (CIMT) of patients [9]. Another study by Jeong et al. found that the serum miR-212 level shows a significant increase in atherosclerosis patients and identified circulating miR-212 as a novel marker of atherosclerosis [7]. The plasma miR-186-5p level is also aberrant in the early stage of acute myocardial infarction (AMI) and there is an evident correlation between the occurrence of AMI and the presence of atherosclerosis [10, 11]. Another study on the role of miRNAs in ascending aortic aneurysm found that miR-186-5p can distinguish aneurysmal tissue from unchanged tissue and is involved in smooth muscle differentiation and contractility [12]. However, its role and underlying mechanism in atherosclerosis have not been reported.

In this study, serum miR-186-5p levels were measured in clinical samples from atherosclerosis patients. Its clinical value and effects on VSMC proliferation and migration were also investigated.

## Materials and methods

### Study population and sample collection

The survey protocol was approved by the Research and Ethics Review Committee of the Yidu Central Hospital of Weifang. It was designed in accordance with the principles of the Declaration of Helsinki. All the recruited individuals signed written informed consent.

The subjects were 104 patients with asymptomatic atherosclerosis and 80 healthy controls. The diagnosis of atherosclerosis was based on the value of the carotid intima-media thickness (CIMT) of the common carotid artery. Cases with CIMT ≥0.9 mm but < 1.2 mm were identified as having asymptomatic atherosclerosis [13]. Demographic and clinical data were recorded. Individuals with a history of smoking, diabetes, cardiovascular and cerebrovascular diseases, cancer, inflammatory diseases or relevant medical treatment were excluded from the study. Fasting blood samples were collected, immediately centrifuged, and then stored at − 80 °C.

### Cell culture and treatment

VSMCs were provided by the American Type Culture Collection (ATCC, USA) and incubated in Dulbecco’s modified Eagle’s medium (DEME; Gibco, USA) containing 10% fetal bovine serum (FBS; PAN, Germany). MiR-186-5p mimic, miR-186-5p inhibitor and their negative controls (mimic NC, inhibitor NC) were purchased form RiboBio (China) and transfected into cells using Lipofectamine 2000 (Invitrogen, USA) according to the manufacturer’s instructions.

### Quantitative real-time PCR

Total RNA was isolated using TRIzol reagent (Invitrogen, USA) and then reverse transcribed into cDNA using PrimeScript RT Reagent Kit (Takara, Japan). Quantitative real-time PCR was carried out to determine the gene expression using a SYBR Premix Ex Taq II Kit (Takara, China). U6 was the internal control and the comparative delta C_T_ (2^−ΔΔCt^) method was applied to calculate the relative gene expression.

### CCK-8 assay

A CCK-8 assay kit (Dojindo, Japan) was used to evaluate cell proliferation. 48 h post-transfection, cells were inoculated into 96-well plates (5 × 10^4^ cells/well). Then the cell viability was analyzed every 24 h by reading the absorbance at 450 nm under an ELx800 absorbance microplate reader (Bio-Tek Instruments, USA).

### Cell Transwell assay

Cell migration was measured using Transwell chambers (Corning, USA). The transfected cells (5 × 10^4^ cells/well) were inoculated into the upper chambers of the inserts in serum-free DMEM and DMEM plus 10% FBS was added into the lower chamber as the attractant. 24 h post-incubation, the cells on the lower membrane were stained using 0.1% crystal violet. Five fields were selected randomly, and the number of cells were counted under an Olympus microscope (Olympus Corporation, Japan).

### Statistical analysis

All data analysis was carried out using SPSS 23.0 software (SPSS, USA) and GraphPad Prism 7.0 software (GraphPad Software, USA). Student’s *t* test was applied for comparisons of two groups, while one-way ANOVA was applied for comparisons of multiple groups. The Spearman correlation coefficient was used to evaluate the correlation between continuous variables. A receiver operating characteristic (ROC) curve was created for the assessment of the predictive power of miR-186-5p for atherosclerosis. *p* < 0.05 indicates a statistically significant difference.

## Results

### Participant characteristics

The participants’ demographic and clinical characteristics are outlined in Table 1. No significant difference was detected between atherosclerosis patients and the control group (*p* > 0.05) for age, gender, body mass index (BMI), total cholesterol, high density lipoprotein cholesterol (HDL-C), low density lipoprotein cholesterol (LDL-C), triglycerides, heart rate, systolic blood pressure (SBP) and diastolic blood pressure (DBP). However, atherosclerosis patients had a significantly higher level of the C-reactive protein (CRP) than the control group (*p* < 0.001).

**Table 1:** Demographic and clinical data for the study population

Features	Healthy controls (*n* = 80)	atherosclerosis patients (*n* = 104)	*p* value
Age (years)	47.98 ± 5.53	48.89 ± 5.06	0.242
Gender (male/female)	40/40	56/48	0.605
BMI (kg/m^2^)	23.49 ± 2.57	23.90 ± 2.69	0.290
Total cholesterol (mg/dl)	189.98 ± 30.53	195.96 ± 26.36	0.156
HDL-C (mg/dl)	52.03 ± 9.08	50.95 ± 7.75	0.389
LDL-C (mg/dl)	126.74 ± 16.74	127.68 ± 21.66	0.739
Triglyceride (mg/dl)	163.03 ± 29.42	173.38 ± 40.04	0.053
Heart rate (beats/min)	76.33 ± 7.10	75.28 ± 6.32	0.293
SBP (mm Hg)	129.91 ± 12.71	129.64 ± 13.68	0.892
DBP (mm Hg)	79.53 ± 7.52	80.93 ± 6.56	0.178
CRP (mg/l)	6.29 ± 1.86	20.87 ± 2.97	0.000
CIMT (mm)	0.45 ± 0.12	1.04 ± 0.07	0.000

### Serum miR-186-5p levels in atherosclerosis patients

**Fig. 1:**
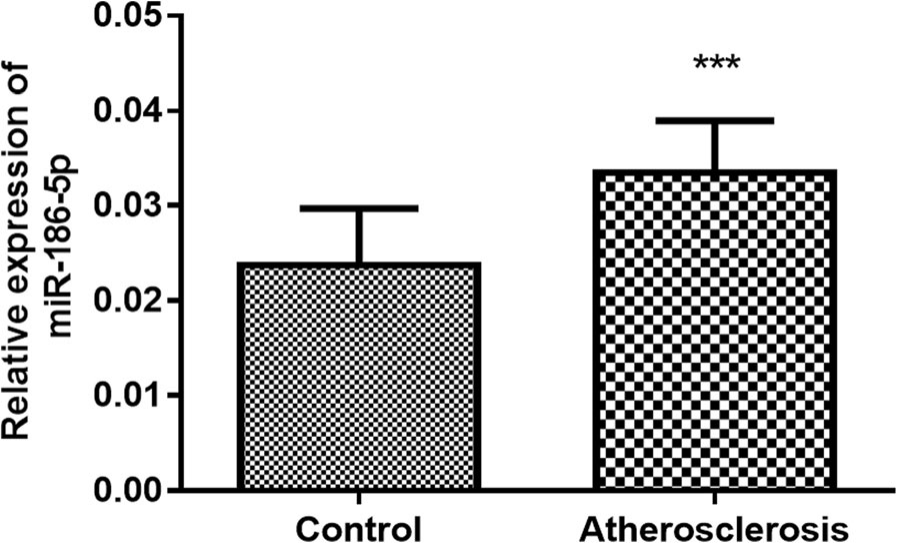
The expression level of miR-186-5p was significantly higher in atherosclerosis patients than in the controls. ***p < 0.001

The miR-186-5p levels in the serum of atherosclerosis patients were measured using quantitative real-time PCR. They had significantly higher miR-186-5p levels than the control group (Fig. 1, *p* < 0.001).

### Correlation of miR-186-5p with CIMT in atherosclerosis patients

**Fig. 2:**
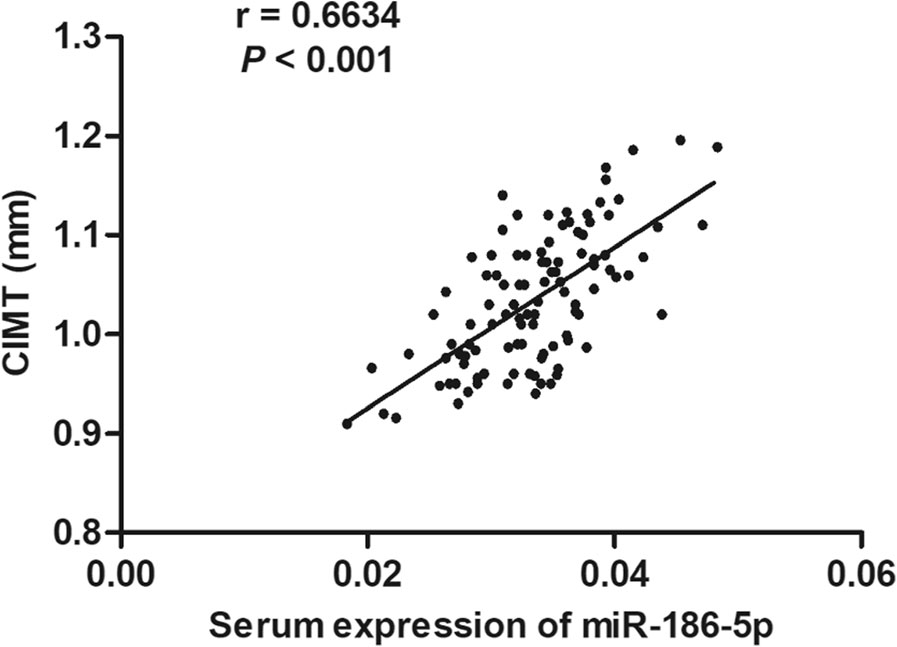
The serum miR-186-5p level shows a positive correlation with CIMT in atherosclerosis patients (*r* = 0.6634, *p* < 0.001)

Carotid intima-media thickness (CIMT) is a well-established predictor for the risk of subclinical atherosclerosis [14]. Here, we found a positive correlation between serum miR-186-5p level and CIMT in atherosclerosis patients (*r* = 0.6634, *p* < 0.001; Fig. 2).

### Specificity and sensitivity of miR-186-5p as a diagnostic biomarker

**Fig. 3:**
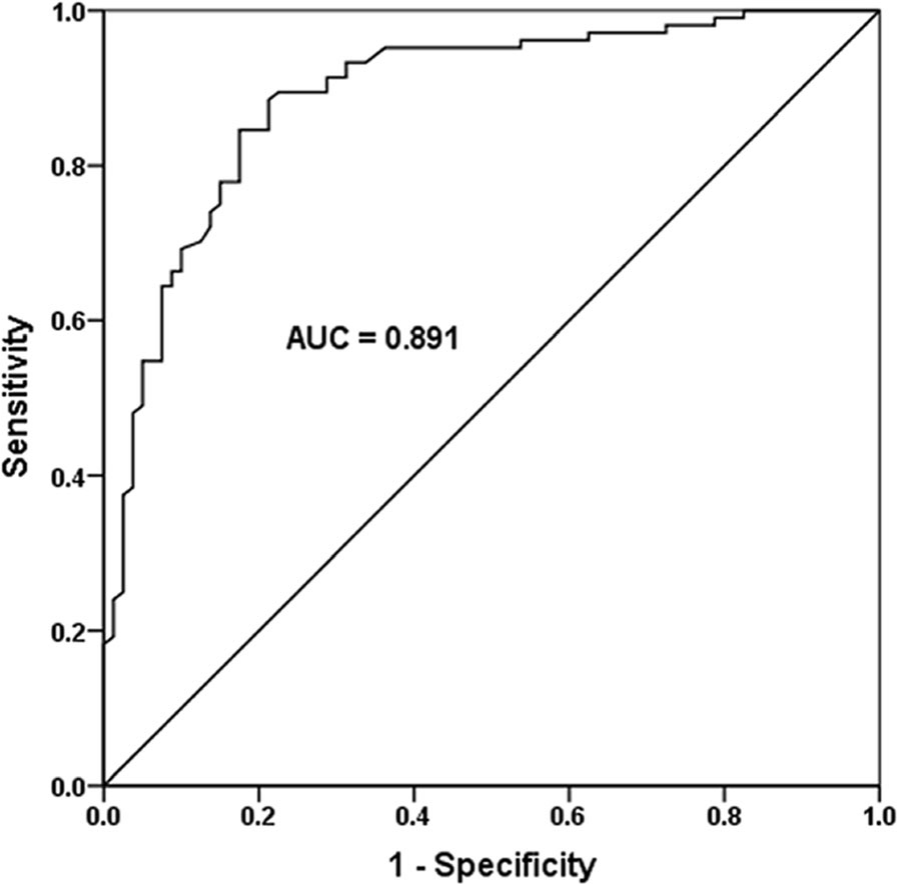
An ROC curve was established to assess the discriminative ability of serum miR-186-5p for identifying atherosclerosis patients from control subjects. A high diagnostic power of miR-186-5p as a biomarker for atherosclerosis was detected (AUC score = 0.891)

An ROC curve was established to assess the discriminative ability of serum miR-186-5p for identifying atherosclerosis from controls. MiR-186-5p had an area under the curve (AUC) score of 0.891 for atherosclerosis (Fig. 3). Based on the sensitivity (88.5%) and specificity (78.7%), a cutoff of 0.027 was determined. The results show that miR-186-5p has the ability to distinguish atherosclerosis patients from healthy controls.

### Effect of miR-186-5p on VSMC proliferation and migration

**Fig. 4:**
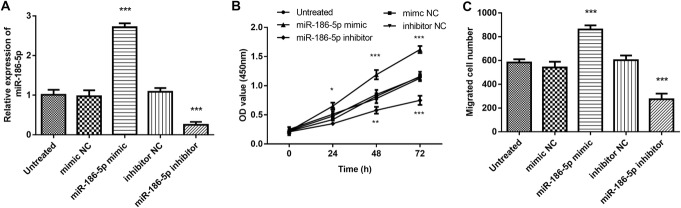
The effect of miR-186-5p on VSMC proliferation and migration. **a** Transfection with miR-186-5p mimics successfully and significantly increased the expression of miR-186-5p. Transfection with miR-186-5p inhibitors successfully and significantly decreased the expression of miR-186-5p. **b** Overexpression of miR-186-5p significantly promoted cell proliferation, while its downregulation yielded the opposite results. **c** The number of migrated cells increased significantly after overexpression of miR-186-5p and decreased after its downregulation. *p < 0.05, **p < 0.01, ***p < 0.001 compared with the untreated group

MiR-186-5p mimics or inhibitors were transfected into VSMCs to regulate the expression of miR-186-5p. Transfection with miR-186-5p mimics successfully and significantly elevated the level of miR-186-5p, whereas miR-186-5p inhibitors successfully and significantly downregulated its expression (Fig. 4a, *p* < 0.001).

The CCK8 assay results show that overexpression of miR-186-5p significantly promoted cell proliferation and its downregulation yielded the opposite results (Fig. 4b, *p* < 0.001). The Transwell assay showed that the number of migrating cells increased significantly after overexpression of miR-186-5p and decreased after its downregulation (Fig. 4c, *p* < 0.001). We concluded that miR-186-5p promotes VSMC proliferation and migration.

## Discussion

The dysregulation of various miRNAs in atherosclerosis has been widely reported [7, 9, 15]. Our study results show an elevated serum miR-186-5p level in atherosclerosis patients, meaning that miR-186-5p might have a function in its occurrence and pathogenesis. Wang et al. determined overexpression of miR-186-5p in AMI and identified circulating miR-186-5p as a promising novel diagnostic biomarker for this condition [10]. Another study proved that the miR-186-5p expression level is significantly elevated in ACS patients and that the high serum miR-186-5p level decreases significantly after percutaneous coronary intervention (PCI) [16]. These results support our findings.

Although it is well known that miRNA can be derived from different cell sources, and finally secreted into blood [17], our results could not clarify the cellular source of miR-186-5p. Further research in this area will help elucidate the underlying mechanism of the effect of miR-186-5p in atherosclerosis.

The clinical value of miRNAs has been widely reported serving as non-invasive biomarkers for assessing disease occurrence and progression, including that of cardiovascular diseases [18, 19]. For example, the level of circulating miR-214 is higher in coronary artery disease (CAD) patients and the level of the increase indicates the severity of CAD. Alternations in the circulating miR-214 level might be a potential marker for the presence of vulnerable plaques [20]. A high expression level of miR-1 has been identified in AMI patients. Plasma miR-1 can be used as a potential biomarker for the early diagnosis of AMI and is correlated with its progression [21].

In our study, an ROC curve was established to evaluate the discriminative ability of serum miR-186-5p for identifying atherosclerosis patients from healthy individuals. Serum miR-186-5p was proved to have a high diagnostic value as a biomarker for atherosclerosis. CIMT is a well-established predictor for atherosclerosis and it contributes to the occurrence of myocardial infarction and stroke [22, 23]. In our study, a positive association was also detected between the serum miR-186-5p level and CIMT in atherosclerosis patients. We concluded that the elevation of circulating miR-186-5p might be an efficient biomarker to predict the occurrence of atherosclerosis.

The clinical value of miR-186-5p in diagnosis and prognosis was also investigated. Dysregulation of circulating of miR-186-5p is considered to be a promising novel diagnostic biomarker for the early phase of AMI [10]. Summerer et al. found that miR-186-5p has a high sensitivity and specificity to classify head and neck squamous cell cancer patients from healthy people, and elevation of serum miR-186-5p is correlated with poor prognosis [24].

Currently, most existing studies have identified that aberrantly expressed miRNAs exert their pathological function by dysregulating normal cell behaviors. As previous studies reported, miR-186-5p has a significant impact on cell growth, migration and invasion in several human diseases. Feng et al. demonstrated that miR-186-5p is involved in the development of lung adenocarcinoma through its promotion of cell proliferation, migration and invasion [25]. Overexpression of serum miR-186-5p is detected in prostate cancer patients and downregulation of miR-186-5p plays an inhibitory role in cell proliferation and invasion [26].

It is well known that the abnormal VSMC behavior is integral to the pathogenesis of atherosclerosis. Therefore, we further investigated the impact of miR-186-5p on the biological function of VSMCs. Overexpression of miR-186-5p was found to significantly promote cell proliferation and migration, whereas a decrease in miR-186-5p had the opposite effect. These results suggest a promoting effect of miR-186-5p on VSMC biological behaviors, which might be the mechanism underlying the role of miR-186-5p in atherosclerosis. Consistently, a major study on the role of miRNAs in ascending aortic aneurysms reported that miR-186-5p is involved in smooth muscle differentiation and contractility, which both suggest a crucial role for miR-186-5p in VSMC behaviors [12].

As previous evidence suggested, phosphatase and tensin homolog (PTEN) is a known target of miR-186-5p [25]. PTEN is a protein–lipid phosphatase that has reportedly low expression in patients with atherosclerosis [27, 28]. Overexpression of PTEN was found to protect against atherosclerosis through inhibition of VSMC proliferation and migration [29, 30]. PTEN is considered to be a negative regulator of PI3K signaling, leading to the inactivation of the AKT pathway [31]. In a study about the role of miR-647 in atherosclerosis, the PTEN/PI3K/AKT pathway was proved to regulate the proliferation and migration of ox-LDL-treated human aorta VSMCs [28].

Considering earlier findings and our current results, we speculate that miR-186-5p might be involved in the initiation and development of atherosclerosis through targeting the PTEN/PI3K/AKT pathway. Although this study provides a novel insight into the clinical and functional role of miR-186-5p in atherosclerosis, more studies are warranted to further clarify the molecular mechanisms. Additionally, our study sample is relatively small. A study with a larger population is needed to confirm our findings.

## Conclusion

We demonstrated that elevated miR-186-5p levels might have a certain diagnostic significance for atherosclerosis and that upregulation of miR-186-5p promotes the VSMC proliferation and migration. This may provide a novel concept for the therapeutic interventions of atherosclerosis.

## Data Availability

All data generated or analyzed during this study are included in this published article.

## Abbreviations

AUC: Area under the curve
miRNAs: MicroRNAs
CIMT: Carotid intima-media thickness
AMI: Acute myocardial infarction
VSMCs: Vascular smooth muscle cells
ACS: Acute coronary syndrome
PCI: Percutaneous coronary intervention
CAD: Coronary artery disease
